# Do Thirty-Second Post-activation Potentiation Exercises Improve the 50-m Freestyle Sprint Performance in Adolescent Swimmers?

**DOI:** 10.3389/fphys.2018.01464

**Published:** 2018-10-22

**Authors:** Zied Abbes, Karim Chamari, Iñigo Mujika, Montassar Tabben, Khalid W. Bibi, Ali Mostafa Hussein, Cyril Martin, Monoem Haddad

**Affiliations:** ^1^Interuniversity Laboratory of Human Movement Biology EA7424, University of Lyon – University Claude Bernard Lyon 1, Lyon, France; ^2^AHP Research Centre, ASPETAR, Qatar Orthopaedic and Sports Medicine Hospital, Doha, Qatar; ^3^Department of Physiology, Faculty of Medicine and Nursing, University of the Basque Country, Leioa, Spain; ^4^Exercise Science Laboratory, School of Kinesiology, Faculty of Medicine, Universidad Finis Terrae, Santiago, Chile; ^5^ASPREV Department, ASPETAR, Qatar Orthopaedic and Sports Medicine Hospital, Doha, Qatar; ^6^Physical Education Program, College of Education, Qatar University, Doha, Qatar; ^7^Sport Science Program, College of Arts and Sciences, Qatar University, Doha, Qatar

**Keywords:** swimming, performance, post-activation potentiation, competition, warm-up, swimmers

## Abstract

**Objectives:** The purpose of the study was to investigate performance, biomechanical, physiological, and psychophysiological effects of a simple and easily organized post-activation potentiation (PAP) re-warm-up performed before a 50-m freestyle swimming sprint.

**Methods:** Regional level male adolescent swimmers [age: 13.0 ± 2.0 years; (min 11 years – max 15 years)] performed four trial conditions (three experimental, one control) on different days. The control trial involved a standardized 1200-m warm-up followed by 30 min of rest and a maximal 50-m freestyle swimming sprint. The experimental trials involved the same protocol but added a PAP component after a 20-min rest (10 min pre-50-m): The different PAP component involved the subjects in completing a 30-s maximal effort of: (1) push-ups (PU – upper body), (2) squats (SQ – lower body), and (3) burpees (BP – lower and upper body). Performance (time-trial), biomechanical (stroke length, stroke frequency), physiological (blood lactate concentrations, heart rate), and psychophysiological (ratings of perceived exertion) variables were collected.

**Results:** The results demonstrated that the PAP protocols used in this investigation had no effect on swimming performance. Before the 50-m swimming sprint, the lactate values were significantly higher after the PU, BP, and SQ PAP loads compared to the control condition [P_(CC-PU)_ = 0.02; P_(CC-BP)_ = 0.01; P_(CC-SQ)_ = 0.04]. For Lactate values, a significant and large effect of experimental condition compared to control condition was found (*p* < 0.05, η^2^ = 0.68). At 1 min after the 50-m time trial, significant differences were observed between the control condition and the different PAP loads [P_(CC-PU)_ = 0.01; P_(CC-BP)_ = 0.04; P_(CC-SQ)_ = 0.01]. At 3 min after the 50-m sprint, significant differences were found between the control condition and the PU and SQ PAP loads [P_(CC-PU)_ = 0.018; P_(CC-SQ)_ = 0.008, respectively]. Additionally, a significant and large effect of experimental condition was found at 1 and 3 min after the 50-m swimming sprint (*p* < 0.05, η^2^_(1 min)_ = 0.73; η^2^_(3 min)_ = 0.59). There were medium sized but non-significant effects of interaction between the conditions, was illustrated for the mean HR values in response to the different conditions (*p* > 0.05; η^2^ = 0.083).

**Conclusion:** None of the three PAP protocols showed any significant improvement in performance, biomechanical, physiological, and psychophysiological variables before, during and after the 50-m swimming time-trial. Further studies are warranted to investigate ways to improve swimming performance with simple body mass exercises performed in-between the end of pool warm-up and race start.

## Introduction

On competition day swimmers have a period of warm-up prior to a race event. The aim of this warm-up period is to help the swimmer optimize psychological, neurological, and physiological states for the best performance (Bishop, 2003). According to Neiva et al. (2014), warm-up has a positive effect on a swimmer’s performance. A follow-up study performed by same author suggested that a 10 min post warm-up passive rest enhances 100 m freestyle performance better than a 20 min passive rest (Neiva et al., 2017). The “Fédération Internationale de Natation” (FINA) rules dictate that swimmers must enter the call room at least 20 min prior to racing (FINA, 2015). This forces swimmers to complete their warm-up at least 30 min before the race. The ergogenic effects of in-pool warm-ups can last up to 20 min but will not endure up to 45 min post-warm-up (West et al., 2013) and it is not possible to re-warm-up in a pool during the last 20 min leading to the race. Hence, alternative forms of re-warm-up are potentially needed. In this context, most studies about warming up in swimming have reported a 10-min period of recovery between the warm-up and the swimming trial, and little is known about swimmer performance when longer recoveries are used (Neiva et al., 2015). In that regards, exercises resulting in post-activation potentiation (PAP) may be an interesting alternative tool to use in between the classical swimming warm-up and the race. PAP is usually denoted in the literature as short bouts of high-intensity exercise that induce fatigue and have the potential to acutely improve the muscle’s capability of generating high forces over a short period of time post-PAP exercise (Sale, 2002). PAP is also known as the induction of the myosin regulatory light chain (RLC) phosphorylation and the potentiation of the isometric twitch force amplitude during maximal or submaximal voluntary contraction of human skeletal muscle (Stull et al., 2011). Stuart et al. (1988) and Grange and Houston (1991) were the first to show that RLC phosphorylation in human skeletal muscle was associated with increased low frequency force or torque output. PAP is also associated with the close interaction between the myosin and actin. This better positioning would favor greater connections among protein filaments and, consequently, greater development of muscle tension (Batista et al., 2010). In addition, it is linked to increased Ca^2+^ concentration in sarcoplasm, implying greater phosphorylation of light-chain myosin, as well as greater formation of cross-bridges (Macintosh et al., 2012). In this sense, the skeletal muscle force output is regulated through Ca^2+^-mediated alterations of the rate at which cross bridges make the transition from non-force-generating to force-generating states (Vandenboom et al., 1993).

However, it is noteworthy that there are mechanisms that determine whether exercise before the main activity will cause muscle strength increase (potentiation) or decrease (fatigue). If the main activity occurs immediately after the conditioning activity, fatigue could predominate over potentiation (Rassier and Macintosh, 2000). The opposite may occur when sufficient interval for muscle recovery is allowed between activities. In this situation, it results in a greater production of muscular power and potentially enhance strength and speed performance (Sale, 2002).

Post-activation potentiation has shown beneficial effects on performance in a wide variety of activities such as maximal voluntary contractions (Chiu et al., 2003), sprint running (Chatzopoulos et al., 2007), and sprinting and jumping (Mola et al., 2014). However, data on the effects of PAP in swimming performance are scant and inconsistent (Hancock et al., 2015; Sarramian et al., 2015; Barbosa et al., 2016). Furthermore, most of those studies implemented PAP tools without the use of weightlifting, such as short sprints with a power rack (Hancock et al., 2015) or hand paddles and parachute (Barbosa et al., 2016). PAP commonly occurs after heavy resistance exercise. However, this method of inducing PAP has limited application to the pre-competition practices (e.g., warm-up) of many athletes and in many activities as swimming. A study conducted by Turner et al. (2015) concluded that sprint performance is enhanced after plyometric exercise provided adequate recovery is given between these activities. In an earlier study, Tobin and Delahunt (2014) examined the acute effect of a plyometric stimulus on jump performance in professional rugby players. The main finding of this study suggest that performing repeated series of plyometric jumps (40 jumps) appears to be an effective method of taking advantage of the PAP phenomenon, thus possibly eliminating the need for a complex training protocol. These findings are encouraging and present a practical method to enhance the pre-competition practices of athletes.

The 50-m freestyle is the shortest and fastest event in Olympic swimming, typically lasting less than 22 s at the elite level and less than 25 s for young elite swimmers. The difference between the first and last swimmers in the finals of international events is often less than 1 s. Given the predominantly anaerobic nature of such a short duration intensive effort (Chamari and Padulo, 2015) and that power through the arm pull in freestyle sprinting is a determinant of swimming speed in elite swimmers (Sharp et al., 1982), PAP could be an interesting method to enhance performance. The use of PAP while in the call room 20 min before the race could give swimmers a competitive advantage, as the effect of the pool warm-up may have dissipated significantly. It was previously shown that PAP could have a beneficial impact on swimming sprint performance (Hancock et al., 2015), but the PAP methods used in the study, i.e., four maximal 10-m swims at a 1-min interval while attached to a resistive power rack, cannot be implemented in a competitive context.

Therefore, the present study aimed to investigate the performance, technical, and physiological effects of a simple and easily organized PAP re-warm-up performed in between the classical swimming warm-up that is finished approximately 30 min before the race and a 50-m freestyle swimming sprint. The PAP loads tested are based on easy bodyweight exercises that can be implemented in a competition context, i.e., in a small area and without the use of any specific equipment.

## Materials and Methods

### Subjects

Seventeen regional level male adolescent swimmers (age: 13.0 ± 2.0 years; height: 161.1 ± 12.4 cm; body mass: 52.5 ± 9.5 kg) with FINA points of 520 pts ± 98, volunteered to participate in the study. To be included in the protocol, participants must have had at least 4 years of experience in competition swimming, training six times per week. They also had to be familiar with performing proper push-ups, squat jumps (SJs), and burpees. The 17 swimmers did not become injured or sick throughout the duration of the study. All participants were informed about the study procedures, requirements, and risks. The participants and their guardians signed an informed consent form prior to the start of the study. The study was conducted according to the Declaration of Helsinki, and it was approved by Qatar University’s Institutional Review Board (QU-IRB 634-A/16) before the beginning of the experiments.

### Experimental Design

The study was conducted in a 50 m competition pool. Data collection took place in February and March during the swimming season, which started in September and ended in June. However, to avoid any effect of competition-related fatigue and/or stress, participants did not take part in any competitive swimming event during the experiment period.

**FIGURE 1:**
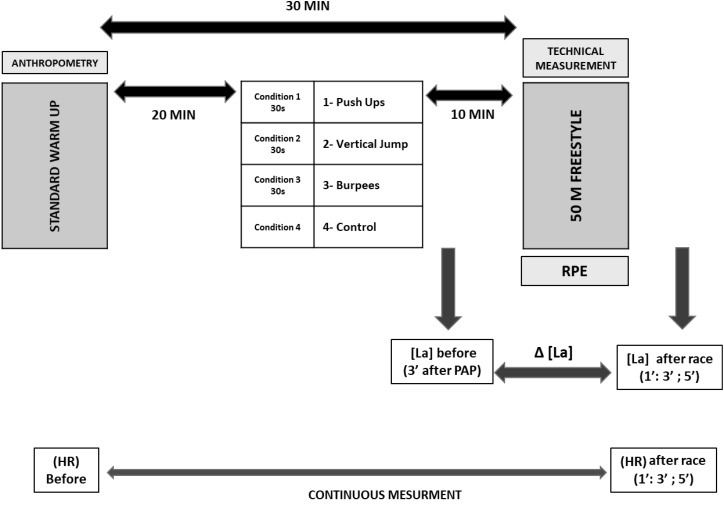
Experimental design. Conditions: Condition 1: 30 s of push-ups, Condition 2: 30 s of vertical jumps, Condition 3: 30 s of burpees, Condition 4: 30 s of control. Sprint warm-up: prepared by the coach using the same conditions as those in competition. Heart rate measured using a water-resistant HR monitor (Polar V800, Polar, Kempele, Finland). Lactate measured using Lactate Scout + (EKF Diagnostics, Cardiff, United Kingdom). Anthropometry: Height, Body mass, Technical measurement: Using a video camera to record the strokes during the swimming race. RPE: According to Borg Scale.

Each swimmer underwent one control session and three experimental sessions separated by 3 days between sessions. The only difference between the four sessions was the re-warm-up modality between the classical warm-up and the simulated swimming race (see Figure 1). Participants were asked to refrain from partaking in any other sport or engage in heavy physical activity for 48 h prior to the experimental sessions. During the 2 days before the experiment, the subjects performed a light aerobic swimming session.

During the experimental sessions, the participants performed a standard warm-up of 1200-m (Neiva et al., 2015) followed by 30 min of rest to simulate the competition warm-up conditions. Within this transition period, the participants were seated in chairs (passive rest). After 20 min, they performed one of the PAP conditions for 30 s (or the control condition, see below), and then rested again for another 10 min before the 50-m sprint. The sprint swim was conducted under race conditions with a dive start. Timing was controlled using a manual stopwatch by the same examiner for all the participants. All swims were recorded using a Panasonic, Full HD, (HC – W570) video camera for later analysis of time and technique. Time measurement (t), stroke rate (SR), and stroke length (SL) were measured using a video camera (Video Analysis).

Stroke rate and SL were calculated using the following equations: (Bielec and Makar, 2010)

• SR = 60 × 3/tSR; where tSR is the time of three cycles. (tSR was measured between 12.5 and 25 m). SR was converted to the international system of unit: Hertz.
• *v* = S/t; where S is the entire distance (50 m), t is time.
• SL (Meter/Cycle) = *v* × 60/SR.

The four intervention conditions (three experimental, one control) were performed in a randomized and partial counterbalanced order. All participants were verbally encouraged during the PAP stimulus. Ratings of Perceived Exertion (RPE) were collected at the end of every 50-m (Borg, 1998; Haddad et al., 2017).

Throughout the experiment, heart rate (HR) values were measured using a water-resistant HR monitor (Polar V800, Polar, Kempele, Finland), which was affixed on the chest of the participant according to manufacturer’s instructions. HR values were extracted 1 min before the 50-m sprint and then 1, 3, and 5 min post sprint. Fingertip blood lactate concentrations were measured 3 min after the PAP and then 1, 3, and 5 min after the 50-m swimming sprint using Lactate Scout+ (EKF Diagnostics, Cardiff, United Kingdom).

### PAP Conditions

#### Condition 1: Upper Body Post-activation Potentiation – Push-ups (PU)

Push-ups are effective at developing upper body strength. The movement consists of pushing up the body weight against gravity which provides a sufficient resistance to develop the Pectoralis Major, Anterior Deltoid, and Serratus Anterior (Dhahbi et al., 2017). Participants were instructed to touch the ground with their chest and fully extend their arms during every movement. Those stimulated muscles are the same muscles used in freestyle swimming (Bishop et al., 2013). In this condition, the maximum number of push-ups conducted with maximum effort was performed for 30 s.

#### Condition 2: Lower Body Post-activation Potentiation – Squat Jumps (SQ)

Squat Jump stimulate lower body muscles, which are a very important element of sprint swimming (Mookerjee et al., 1995; West et al., 2011). Participants performed the maximal number of vertical SJs with maximum effort for 30 s. The participants started with their feet about shoulder width apart, lowered their body by bending the knees and moving the buttocks backward, and kept the back straight, eyes ahead and knees over the toes. The participants jumped straight up as high as they could with free arm movement.

#### Condition 3: Combined Post-activation Potentiation – Burpees (BP)

Combined PAP consisted of performing the maximum number of burpees for 30 s. The burpee is a full body exercise used in strength training as high intensity exercise. The basic movement is performed in four steps and is known as a “four-count burpee.” The subjects start in a standing position, drop into a squat position with the hands on the ground, kick the feet back while keeping the arms extended, do a push up, return the feet immediately to the squat position and jump up to finish in the standing starting position.

#### Condition 4: No Post-activation Potentiation – Control

During the entire 30-min rest interval, participants sat in chairs and waiting for the 50-m maximal swimming.

### Statistical Analysis

Data are expressed as the means ± standard deviations (SD). Before using parametric tests, the assumption of normality was verified using the Shapiro–Wilk test of normality. A two-way analysis of variance (ANOVA) for repeated measures was used to examine the differences between the four conditions (push-ups, SJs, burpees, and control) for the different variables: 50-m swimming performance, blood lactate, and HR, over the different time.

A one-way analysis of variance for repeated measures was used to compare the means of the various samples (using the F distribution): 50-m swimming time, SR, SL, and RPE between the four conditions (4). Sphericity was checked by the Mauchly test. When the assumption of sphericity was not met, the significance of the F-ratios was adjusted according to the Greenhouse-Geisser procedure. The Bonferroni *post hoc* test was used to identify significant differences. The magnitude of these differences was assessed by the effect size (η^2^). According to Cohen (1988), the magnitude of effect sizes (η^2^) can be classified as small (0.01 ≤ η^2^ < 0.06), medium (0.06 ≤ η^2^ < 0.14), and large (η^2^ ≥ 0.14) (Cohen, 1988). This effect size can only measure the main effect. Statistical significance was set a-priori at *p* ≤ 0.05, and all analyses were performed with Statistical Package for the Social Sciences (SPSS) software (release 23, Armonk, NY, United States).

## Results

As shown in Table 1, the ANOVA results revealed that the PAP re-warm-up protocols had no significant effect on the 50-m swimming performance, SR, SL, or post-exercise RPE.

**Table 1:** Effects of the four post-activation potentiation conditions on 50-m freestyle swimming performance: (i) stroke rate, (ii) stroke length, post-exercise rate of perceived exertion (RPE), blood lactate concentrations [La] and heart rate.

	Performance (s)	Stroke rate (Hz)	Stroke length meter/cycle)	RPE	[La] (mM/L)				Heart rate - (bpm)			
						
					Pre-50-m	1 min post-	3 min post-	5 min post-	Pre-50-m	1 min post-	3 min post-	5 min post-
PU (*N* = 17)	32.62 ± 2.81	0.8 ± 0.08	1.93 ± 0.25	8.3 ± 1.5	3.6 ± 0.9^∗$^	7.0 ± 1.8^∗#^	8.5 ± 2.1^∗#^	8.6 ± 2.3^#^	86 ± 18	130 ± 15	99 ± 13	97 ± 11
SQ (*N* = 17)	32.42 ± 2.32	0.81 ± 0.08	1.92 ± 0.24	8.5 ± 1.0	2.9 ± 1.3	7.3 ± 1.9^∗#^	8.5 ± 2.1^∗#^	8.2 ± 1.8^#^	85.08 ± 17	136 ± 11	105 ± 18	95 ± 18
BP (*N* = 17)	32.46 ± 2.26	0.8 ± 0.08	1.94 ± 0.22	8.0 ± 2.0	3.1 ± 0.7^∗^	7.5 ± 2.2^∗#^	8.4 ± 2.5^#^	8.3 ± 2.2^#^	86.91 ± 21	136 ± 19	103 ± 17	99 ± 15
Control (*N* = 17)	32.84 ± 2.53	0.79 ± 0.07	1.96 ± 0.20	8.0 ± 1.0	1.9 ± 0.8	5.3 ± 1.5^#^	6.8 ± 1.9^#^	7.1 ± 2.0^#^	83 ± 18	125 ± 24	97 ± 23	104 ± 17


*Means ± SD; PU, push-ups; SQ, squat jumps; BP, burpees; RPE, rating of perceived exertion; blood Lactate concentration [La]; Heart rate (HR). ^∗^Significantly different from control (*p* < 0.05), ^$^Significantly different from SQ (*p* < 0.05), ^#^Significantly different from pre-50-m sprint (*p* < 0.05).*

Before the 50-m race, the lactate values were significantly higher after the PU, BP, and SQ PAP loads compared to the control condition [P_(CC-PU)_ = 0.02; P_(CC-BP)_ = 0.01; P_(CC-SQ)_ = 0.04]. For Lactate values, a significant and large effect of experimental condition compared to control condition was found (*p* ≤ 0.05, η^2^ = 0.68). At 1 min after the 50-m time trial, significant differences were observed between the control condition and the different PAP loads [P_(CC-PU)_ = 0.01; P_(CC-BP)_ = 0.04; P_(CC-SQ)_ = 0.01]. At 3 min after the 50-m sprint, significant differences were found between the control condition and the PU and SQ PAP loads [P_(CC-PU)_ = 0.018; P_(CC-SQ)_ = 0.008, respectively]. Additionally, a significant and large effect of experimental condition was found at 1 and 3 min after the 50-m swimming sprint (*p* ≤ 0.05, η^2^_(1 min)_ = 0.73; η^2^_(3 min)_ = 0.59).

There were medium sized but non-significant effects of interaction between the conditions, was illustrated for the mean HR values in response to the different conditions (*p* > 0.05; η^2^ = 0.083).

## Discussion

The purpose of this study was to investigate the performance, technical, and physiological effects of a simple and easily organized PAP re-warm-up performed in between the classical swimming warm-up and a 50-m freestyle swimming sprint. The results showed that the 30-s PAP exercises used in this investigation 10 min before the 50-m race had no effect on sprint swimming performance.

The PAP load implemented in this study included a lower and/or upper body stimulus. A previous study by Gollhofer et al. (1998) found that performing high-intensity maximal voluntary contractions caused a twitch tension that produced a short-term increase in explosive force in both the upper and lower body. The PAP exercises performed in the present study did not improve the 50-m freestyle times of the swimmers. In line with this study, Sarramian et al. (2015) examined the effects of four different warm-ups (a traditional race-specific warm-up, upper body PAP, lower body PAP, and combined PAP warm-up) on the 50-m freestyle swimming performance of elite swimmers using three maximum repetitions of pull-ups (upper body) and five jumps on a box while carrying 10% of their body weight (lower body). As in the present study, the overall results did not show any beneficial effects of PAP compared to those of the traditional race-specific warm-up. On the other hand, the authors suggested that an individualized PAP warm-up may be a valuable tool to enhance the performance in sprint events.

According to Macintosh et al. (2012), the ideal time for potentiation is from 1 to 5 min because this is the time in which light-chain myosin remains phosphorylated, creating, according to the authors, a contraction “memory.” Beyond this period, this memory is dissipated, and potentiation is impaired. In another study, Vandenboom (2016) mentioned that the myosin RLC phosphorylation may represent a form of thick filament activation that provides a “molecular memory” of contraction. Gittings et al. (2017), in another hand, concluded that concentric force potentiation was highly speed dependent. However, the meta-analysis study of Wilson et al. (2013), based on 32 primary studies, concluded that the potentiation was optimal after multiple (vs. single) sets, performed at moderate intensities, and using moderate rest periods lengths between 7 and 10 min. Nevertheless, the 10-min recovery time used in this study may have been too long to induce a PAP effect; however, according to Mettler and Griffin (2012), the simultaneous existence of potentiation and fatigue makes it difficult to determine the point where fatigue declines and potentiation occurs. The present study corroborates more the 5 min interval proposed by Macintosh et al. (2012) as we did not observe any improvement in our experiment. On the other hand, the relatively low load of our chosen tasks may also have been the reason of lack of potentiation effect (too short exercise and/or of too low intensity). Zhi et al. (2005) showed that neither brief tetanic stimulation nor repetitive low frequency stimulation increased RLC phosphorylation. Therefore, an individualized resting time would eventually have resulted in greater potentiation effects, but this remain purely speculative. This hypothesis would be in line with previous studies suggesting the PAP response be clearly individualized (Kilduff et al., 2008; Stone et al., 2008). A study conducted by Stone et al. (2008) demonstrated that a 2-min rest period is sufficient to allow the fatiguing effects of the loading protocol to subside and provide a performance increase. Kilduff et al. (2011) used a squat protocol to observe a PAP effect for sprint swimmers during the start of a sprint race, in the only study to directly examine the immediate effect of PAP on swimming performance. All subjects showed the greatest PAP response (measured by CMJ performance) at 8 min following the loading stimulus, and as such this was the rest interval applied to the swimming trials in their study. In another study, Kilduff et al. (2008) examined CMJ performance following bouts of heavy exercise at different recovery intervals compared with a baseline trial. The results of the study showed that 8 min of recovery time produced the optimum performance following a heavy weight lifting protocol, with a statistically significant performance improvement over baseline occurring after this time interval. Twelve of the subjects (70%) achieved their best measures for jump height, power output, and peak RFD during the 8 min trial condition. Three more participants attained their peak measures at 12 min, while the other three attained their peak earlier (at 4 min post-PAP). There was a reduction in CMJ performance during the trial immediately (∼15 s post-PAP) following the squat protocol, demonstrating that fatigue outweighed potentiation immediately following the loading protocol. In this study we didn’t focus on individual PAP response. Therefore, in future studies, the duration of the recovery period after PAP loading should probably be individualized according to each participant in an attempt to achieve optimal performance. But, before considering individualization, one has to already set a PAP protocol that brings improvement to performance, which hasn’t been achieved by the present study.

In general, the absence of significant results is associated with inadequate type, duration and intensity of the conditioning activity (PAP) (Kilduff et al., 2007; Lim and Kong, 2013). A second potential explanation for the lack of a performance enhancing effect is that the load of the 30-s PAP used in this investigation was not enough to create a potentiation effect. This may be associated with neuromuscular and molecular changes induced by strength and power activities such as: (1) better recruitment of motor units, especially type-IIx motor units, which have a higher contraction velocity and capacity to generate tension; (2) increased availability of Ca^++^ for contraction; and (3) increased phosphorylation of light myosin chains, facilitating the interaction of contractile filament (Batista et al., 2010; Macintosh et al., 2012). These mechanisms are more effective in type-IIx units, which help explaining the manifestation of PAP in trained athletes (Xenofondos et al., 2010). Therefore, the short duration of the PAP probably did not evoke a significant intensity in the muscle and therefore did not induce enough potentiation (Fukutani et al., 2014).

The relationship between SR, SL speed (V) and performance is one of the major points of interests in biomechanical research. Increases or decreases in v are determined by combined increases or decreases in SF and SL, respectively (Barbosa et al., 2008). This study examined several biomechanical markers related to sprint performance during a maximal 50-m swimming. No significant variation was observed in the SR or SL between the four conditions (PU, SQ, BP, CC). This finding can be due to several factors modulating the magnitude of a given PAP response to the pre-load: the young age of our swimmers (13.0 ± 2.0 years), their short training experience and level of swimming, the rest period between the PAP and the 50-m swimming, and/or the volume and relative intensity of the PAP stimulus prescribed (Chatzopoulos et al., 2007).

In this study, the RPE values did not differ between the conditions, indicating that the perception of exercise difficulty was independent of the PAP condition and did not appear to impact the post-effort RPE.

Similar 50-m HR values were observed for the different conditions. This result could probably be due to the long recovery period post-PAP. On another note, according to some authors, the coexistence of fatigue and PAP is relative, and a plausible reason for the unclear results might be the training status of the subjects (Brandenburg, 2005). Athletes who are used to dryland training may have an improved intramuscular coordination for higher loads (Mahlfeld et al., 2004) and, therefore, may be less likely to benefit from the PAP loads.

Despite, the young age of the participant swimmers, no age-related differences were obtained in PAP relative to body mass (Paasuke et al., 2000). Arabatzi et al. (2014) examined the PAP effects on SJ performance in preadolescent (10–12 years), adolescents (14–15 years), and adults (20–25 years) males and females. The study suggested that PAP effects on SJ performance is age- and sex-dependent; that is PAP appears as a viable method for acutely enhancing SJ performance in men but not in pediatric population. But in another study, by Paasuke et al. (2000), No age-related differences were obtained in PAP, rest and potentiated twitch contraction and half-relaxation time, to body mass. Therefore, future studies need to focus more on the effect of PAP on age.

The protocol of the present study does not allow the identification of the factors that potentially led to the lack of improvement in the 50-m sprint: the 10-min lapse between the PAP exercise and performance and/or the load/intensity of the PAP. Further studies are warranted to investigate ways to improve swimming performance with simple body mass exercises performed in-between the end of pool warm-up and swimming performance.

### Study Limitation

This study has some limitations. In general, no study evaluating the effects of conditioning activities in a competitive situation has been found in literature.

As mentioned previously, this study was implemented on young swimmers (preadolescent and adolescents) with short training experience. This could be interpreted as a limitation.

## Conclusion

This study demonstrated that 30-s PAP exercises performed 10 min prior to the event did not impact the 50-m freestyle sprint performance in young swimmers. Extensive research exists examining the PAP effect after a heavy resistance exercise. However, there is limited research examining the PAP effect after a plyometric stimulus. Further research is needed to continue exploring this important scope in sports performance especially in swimming which remains controversial and relatively unknown.

## Author Contributions

All authors contributed to the data analysis and interpretation of the data, drafting, and revising the manuscript, and approved the final version of the manuscript. The original study design was made by MH, KC, CM, ZA and discussed with the other authors. MT, MH, and ZA performed the data analysis.

## Conflict of Interest Statement

The authors declare that the research was conducted in the absence of any commercial or financial relationships that could be construed as a potential conflict of interest. The handling Editor declared a past co-authorship with one of the authors KC.
